# Survival of dental implants placed in autogenous bone grafts and bone flaps in head and neck oncology patients: a systematic review

**DOI:** 10.1186/s40729-018-0131-9

**Published:** 2018-07-04

**Authors:** Dominic P. Laverty, Robert Kelly, Owen Addison

**Affiliations:** 1 Birmingham Community Healthcare NHS Foundation Trust, Birmingham, B5 7EG UK; 2 University of Birmingham School of Dentistry, 5 Mill Pool Way, Birmingham, B5 7EG UK; 3 School of Dentistry, University of Alberta, Edmonton, AB T6G 1C9 Canada

**Keywords:** Dental implants, Autogenous bone graft, Head and neck oncology, Implant survival

## Abstract

Using implants to retain prostheses as part of the oral rehabilitation of head and neck cancer patients is an increasingly common treatment modality, particularly in transported bone which is used to reconstruct defects following oncological surgical resection. The aim of this systematic review is to evaluate the survival of dental implants placed into autogenous bone grafts and flaps, in head and neck cancer patients. MEDLINE, EMBASE, CENTRAL and Science Direct databases were searched (1980-August 2017) for studies evaluating intra-oral implant placement into autogenous bone grafts and flaps in H&N cancer patients. Twenty articles were included reporting on 1905 implants placed into autogenous bone in head and neck cancer patients. Implant survival varied from 54 to 100% within the studies with 11 studies reporting implant survival of over 90%. In conclusion, intra-oral implant survival in autogenous bone grafts in head and neck oncology patients is promising, however inconsistencies in data reporting and in outcome definitions precludes formal meta-analysis.

## Review

### Introduction

#### Rationale

The use of implants to retain prostheses as part of oral and dental rehabilitation of head and neck (H&N) cancer patients is becoming an increasingly common treatment approach [1–3]. A number of benefits advocating implant anchorage over conventionally secured prostheses have been proposed [4] but importantly include a significant improvement in the reported quality of life (QoL) of patients [5].

Patients with H&N cancer often undergo ablative surgery with or without surgical reconstruction, radiotherapy and chemotherapy [4, 6]. Both surgical and non-surgical interventions can lead to significant disability, including facial deformity, loss of hard and soft tissue, impaired speech, swallowing and mastication [7]. Oral and dental rehabilitation has conventionally required the use of removable prostheses to obturate defects, to replace missing tissue structures and to restore function and aesthetics. In this patient group, removable prostheses are often poorly tolerated, are difficult for the patient to maintain and frequently fail to fully achieve the intended functional improvement. The use of dental implants has been proposed to enable secure anchorage for prostheses, reduced loading on vulnerable tissues and provide a better functional and cosmetic solution [8].

However, dental implants can only be placed if there is sufficient bone to encase the implant so that a direct interface between the implant surface and bone can be achieved. Frequently following resective surgery, insufficient bone volume remains and bony reconstruction of the surgical defect is required to enable successful dental implant placement [9]. Patients are commonly reconstructed with either a non-vascularised bone graft or a composite free flap. A non-vascularised bone graft is a free piece of non-vascularised bone (or bone substitute) that is placed in the tissues. A free flap is a vascularised piece of bone (pedicled), which is being increasingly used to reconstruct tumour patients.

High ‘survival’ and ‘success’ rates have been reported in the literature for dental implants placed into autogenous bone grafts in healthy patients but notably the success rates remain lower for implants placed into healthy native bone [10, 11]. With the increasing use of complex reconstructive techniques in rehabilitation following H&N cancer and the placement of dental implants into transported bone, there is a need to appraise the highly varied evidence that is currently available in order to help inform clinical decision making.

#### Objectives

It is the aim of this systematic review to evaluate the survival of dental implants placed into autogenous bone grafts, in H&N oncology patients.

## Methods

### Protocol

The Preferred Reporting Items for Systematic Reviews and Meta-Analyses (PRISMA) [12, 13] for describing and summarising the results of our review was used [12, 13].

A quality assessment of all selected full-text articles was performed using the Methodological Index for Non-Randomized Studies (MINORS) [14] assessment tool to assess the risk of bias of the included studies. The MINORS scoring list consists of 12 items, eight apply to non-comparative studies, and a further four apply to comparative studies. Items are scored as 0 (not reported), 1 (reported but inadequate), and 2 (reported and adequate) with this then totalled up to give a score with the higher scores representing a reduced risk of bias [14]. This was chosen over the Cochrane collaborations’ tool for assessing risk of bias for randomised controlled studies since none of the studies included were randomised control trials.

### Eligibility criteria

#### Inclusion criteria

Studies that met the following criteria where included:

1. Dental implant placement into patients with cancer of the H&N.
2. Dental implants placed into autogenous bone grafts.
3. Studies performed on humans.
4. Patients over 18 years old, or if there are patients under 18 years old within the study that these patients and their data can be removed from the analysis.
5. English language articles.
6. Any study design reporting on at least 35 dental implants or 20 patients who have had implants placed into autogenous bone.
7. Data related to implant number and implant survival in autogenous bone grafts that was either directly reported or can be calculated from data within the study.

#### Exclusion criteria

Studies were excluded if they met the following criteria:

1. Studies that reported on craniofacial or extra-oral implants only.
2. No reported implant survival or an inability to calculate implant number or survival from reported data.
3. Studies reporting on patients under 18 years old where there no ability to remove these patients and their data from the analysis.
4. Laboratory or animal-based studies.
5. Studies with less than 20 patients or 35 dental implants placed into autogenous bone grafts.
6. Review articles.

### Information sources

Four electronic databases were used to systematically search the available literature: (1) The National Library of Medicine (MEDLINE via PubMed), (2) EMBASE, (3) Cochrane Central Register of Controlled Trials and (4) Science Direct. The searches were limited to studies involving human subjects and publication dates from January 1980 to August 2017 that satisfied the inclusion criteria.

### Search

The following search terms were used: *Population*: (<[text words] dental implant OR dental implant* OR oral implant OR oral implants OR osseointegrated implants OR endosseous implant OR dental implantation <[MeSH terms/all subheadings] AND (<[text words] head neck OR squamous cell carcinoma OR oncology OR tumour OR cancer OR malignant OR neoplasm <[MeSH terms/all subheadings] AND *Intervention*: free flap OR vascularized flap OR hard tissue graft OR micro vascularized flap OR micro anastomosed flap OR anastomosed flap OR native bone OR DCIA OR deep circumflex iliac artery OR radial OR scapula OR fibula OR iliac OR rib OR costochondral <[MeSH terms/all subheadings].

### Study selection

Two reviewers (DL and RK) carried out the primary search by screening independently the titles and abstracts and identifying the studies appearing to meet the inclusion criteria. Studies with insufficient information in the title and abstract to make a clear decision were identified and the full paper was reviewed. Those studies selected for evaluation of the full manuscript were carried out independently by the same reviewers who determined the final inclusion. Any disagreement was resolved by discussion with a third independent reviewer (OA). The reasons for rejecting studies at this or subsequent stages were recorded.

### Data collection process

Two reviewers (DL and RK) then independently extracted the data using a bespoke data extraction form. Any disagreement was resolved by discussion with a third reviewer (OA). Studies with missing or incomplete data were excluded and reference lists of the selected studies were checked for cross-references to search for papers that might meet the eligibility criteria for inclusion.

### Data items

Data was collected for implant survival, implant success, implant failure, implant complications, surgical implant placement protocol, implant system used, clinical follow-up, how the author defined success/survival, the type of autogenous bone graft, implant site, the prosthodontic rehabilitation and type of cancer, and the use of radiotherapy were documented where possible.

### Risk of bias in individual studies

A quality assessment of all selected full-text articles was performed using the Methodological Index for Non-Randomised Studies (MINORS) [14] assessment tool.

### Summary measures

The main outcome measure was implant survival. This review will define implant survival as an implant still in situ that has not been removed or lost at the census date and thus implant failure defined as an implant that has been removed or lost and is no longer in situ.

### Synthesis of results

The survival and success figures documented where possible are taken directly from the study; however, where the study did not specifically document the survival or success of implants placed into autogenous bone as a percentage, this was calculated from the data provided (as a function of surviving or successful implants from total reported as placed), and studies that lacked data to calculate this were rejected as part of the secondary screening process.

### Additional analyses

No further analyse was carried out.

## Results

### Study selection

**Fig. 1:**
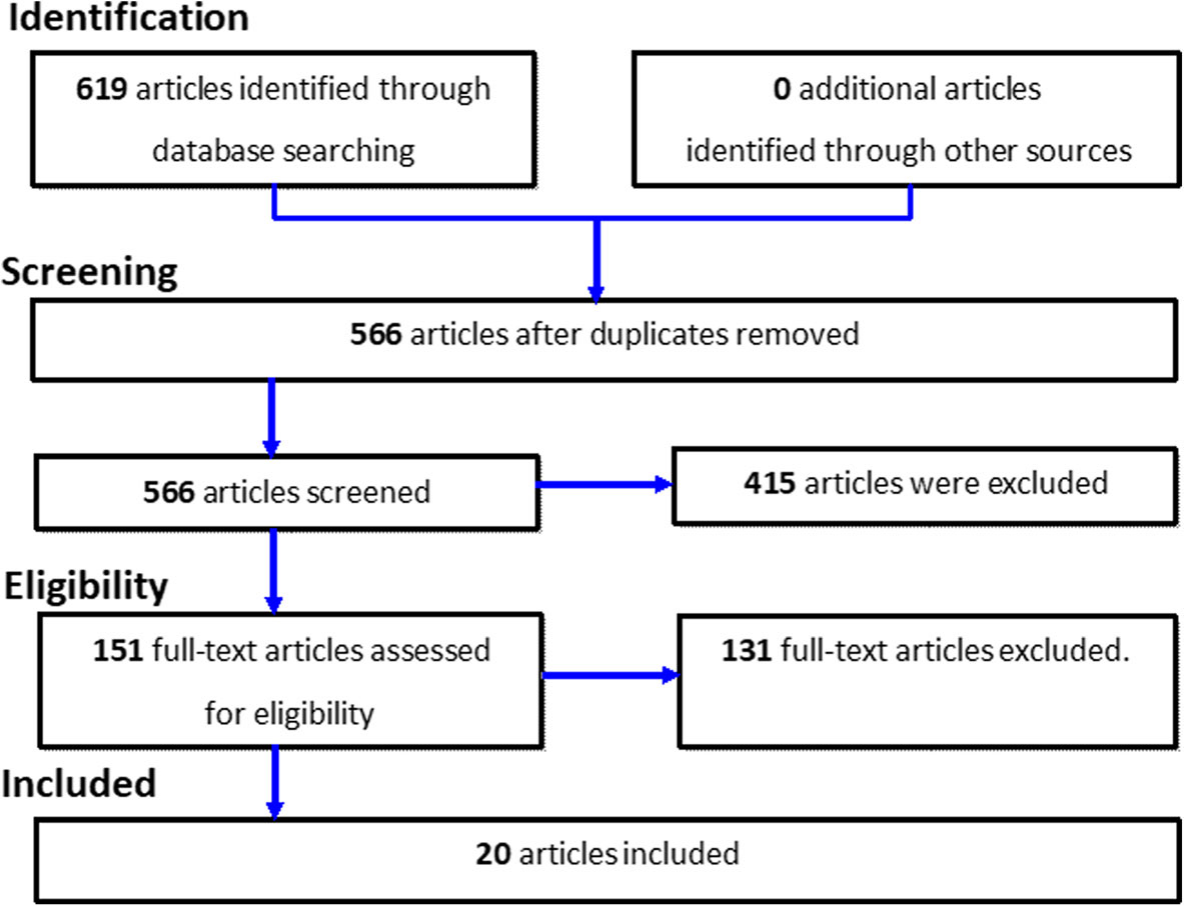
Flow chart of study selection procedure

Searches of EMBASE, the Cochrane Central Register of Controlled Trials, Science Direct and MEDLINE generated 619 articles. After duplicate articles were removed, 566 unique articles were remaining. After the review of the titles and abstracts, 151 articles were accepted for further consideration, and 415 were rejected. After the full text was attained and reviewed for the 151 articles, 131 articles were rejected leaving 20 articles to be included in the systematic review (Fig. 1).

### Study characteristics

The following data was extracted from the studies; study design, centres (single vs multiple centres), patient demographics (patient age, H&N cancer diagnosis), treatment modalities (surgery, radiotherapy, chemotherapy), donor site of autogenous bone graft, outcome measures, implant details (implant system, implant number, implant site, type of bone implant placed into (non-vascularised vs vascularised/free flap), implant placement surgical protocol implant survival/success/failure figures), implant definitions (implant survival/success/failure), type of prosthetic rehabilitation (fixed vs removable), and any reported complications.

### Risk of bias within studies

There were varying scores attained by the studies using the MINORS assessment tool, ranging from 7/16 to 13/16 representing varying degrees of bias within the studies (Table 1).

**Table 1:** Study characteristics and MINORS scores

Author	Year of publication	Study design	Outcome measure	Criteria—survival	Criteria—success	Quality assessment using the MINORS assessment tool	Head and neck cancer diagnosis	Patients age range	Follow-up period	Implant site	Implant system	Implant placement protocol	Prosthodontic rehabilitation
Studies with an average follow-up of 3 years or greater													
Watzinger et al. [29]	1996	Retrospective observational	Implant survival in irradiated mandibles and Outcomes of Peri-implant bone	Not defined	N/A	7/16	SCC	Range = 41–79 years	Up to 3 years	Mandible	IMZ	Primary and secondary implant placement. Secondary placement 6 months after oncological reconstruction. Delayed loading of implants of at least 6 months.	Removable
Teoh et al. [26]	2005	Retrospective observational	Implant survival in the reconstructed mandible and prognostic factors.	Own—implant not removed then survived.	N/A	12/16	SCC, Osteogenic sarcoma, Benign tumours, mucoepidermoid carcinoma and other sarcomas	Mean = 42 years (range = 67–80.5 years)	Mean = 51.7 months (range = 1.3–138 months)	Mandible	Nobel and Osseotite	Delayed loading of implants 6 months after placement. Fixations screws removed prior to implant placement	Fixed and removable
Wu et al. [30]	2008	Retrospective observational	Clinical outcomes of dental implants placed in fibula free flaps for orofacial reconstruction	Own—implants still functioning with no mobility, pain or infection, but with peri-implant bone resorption more than 2 mm were classified as survived.	Albrektsson et al.1986	9/16	Benign and malignant head and neck tumours	Average 47.1 years	Average 47.8 months	Maxilla and mandible	ITI and Branemark	19 patients had primary implant placement 10 patients had secondary placement after oncological reconstruction. Delayed loading of implants of at least 3 months after placement.	Fixed and removable
Fenlon et al. [19]	2012	Retrospective observational	Implant survival	Poorly defined—implant osseointegrated and in situ then survived (usefulness of implant assessed using own 4-point index)	N/A	12/16	Cancer	Unknown	At least 3 years	Unknown	Nobel Biocare, Endopore, Astra and unknown implants	95 implants were primarily placed and 50 implants had secondary placement 3 months after oncological reconstruction.	Unknown
Ch’ng et al. [20]	2014	Retrospective observational	Implant survival, assess effect of risk factors associated with poor healing.	N/A	Own—implant success was defined as a painless and stable fixture without evidence of peri-implant infection or radiographic lack of osseointegration	12/16	SCC, recurrence, osteosarcoma, desmoid tumour, adenoid cystic carcinoma, adenocarcinoma, fibrosarcoma, melanoma, MEC, hemangioma-endotheiloma	Median age = 59 years	Mean = 3.1 years	Unknown	Astra	Primary and secondary implant placement. Patients had implants placed prior to radiotherapy. Reconstruction plates and screws removed if hindering implant placement. Debulking of soft tissues and vestibuloplasty also carried out as required.	All removable
Shaw et al. [31]	2005	Retrospective observational	Implant survival and complications and surgical complications	N/A	Own—implant success was defined as remaining function, no mobility, pain or infection.	10/16	80% of patients SCC, other 20% unknown	Mean = 58 year (range = 15–80 years)	Mean = 3.5 years (range = 0.3/14 years)	Maxilla and mandible	Frialit II, IMZ, Branemark, and IMTEC	Secondary implant placement 1 year after oncological reconstruction. Delayed loading of implants of 3–6 months. Debulking of soft tissue and mucosal grafts carried out as required.	Fixed and removable
Wang et al. [21]	2015	Retrospective observational	Vertical bone Height—double barrel vs vertical distraction Osteogenesis in Fibula Free Flaps, Implant Survival and Success	Poorly defined - implant still in situ then survived.	Albrektsson et al.1986	12/16	Ameloblastoma and OKC	Range = 28–55 years	Mean = 42.5 months ± 4 months	Mandible	Straumann	Secondary implant placement after oncological reconstruction. Delayed loading of implants 3-5 months after placement. Distraction osteogenesis devices used as implants and restored.	All fixed
Yerit et al. [16]	2006	Retrospective observational	Implant survival in the mandible after radiotherapy and radical surgery in oral cancer patients.	N/A	Own - Implant Success when no complaints of the patient, no mobility, no peri-implant tissue inflammation and no peri-implant bone loss exceeding one-third of implant length was observed	10/16	Cancer of oral cavity (majority of the subjects having destructive oral squamous cell carcinomas stage T2–T4)	Range = 16–84.1 years	Mean = 5.42 years ± 3.21 years	Mandible	IMZ, Frialit II and Xive	Implant insertion at various intervals with the mean at 1.41 years after reconstruction. Delayed loading of implants of at least 6 months. Gingivoplasty and vestibuloplaty procedures carried out as required.	Removable
Linsen et al. [17]	2009	Retrospective observational	Survival of implants and implant-retained prostheses in patients after ablative surgery of oral cancer with or without adjunctive radiation therapy.	N/A	Kaplan et al. 1958	9/16	SCC, Ameloblastoma, Adenoid Cystic Carcinoma, OKC, Carcinoma of other origins	Mean = 55.7 year (range = ± 16.25 years)	Mean = 47.99 months ± 134.31 months)	unknown	Branemark and Straumann	Delayed implant placement with an average of 41 months after oncological treatment. Delayed loading of implants of 4.9 months (average).	Fixed and removable
Studies with an average follow-up of less than 3 years or no average follow-up reported													
Fierz et al. [25]	2013	Retrospective observational	Reports on surgical and prosthodontic rehabilitation after resection for oral oncology resection	Own—implant not removed then survived, those functioning given a ‘survival rating’	N/A	9/16	SCC, Adenocarcinoma and Others tumours	Mean = 57 year (range = ± 7.2 years)	Range = Less than 12 months up to 5 years	Maxilla and Mandible	Unknown	No described protocol.	Fixed and removable
Barrowman et al. [7]	2011	Retrospective observational	Audit experience of implant placement in jaws after oral cancer resection, Success of Prosthodontic Rehabilitation	Poorly defined - implant still in situ then survived	N/A	10/16	SCC, Verrucous Carcinoma, Osteosarcoma and Adenoid Cystic Carcinoma	Range = 20–76 years	Up to 15 years	Maxilla and Mandible	Branemark	No described protocol.	Fixed and removable
Zou et al. [22]	2013	Retrospective observational	Long-term clinical outcomes on immediate or staged Implant Placement in iliac bone for restoring defects after tumour resection.	Own - Implants provided supportive function and were stable when torque tested	Albrektsson et al.1986	7/16	SCC, Ameloblastoma, OKC, Myxoma	Range = 24–61 years	Up to 12 years	Mandible	Nobel and Straumann	17 patients had primary implant placement 15 patients had secondary placement after oncological reconstruction. Delayed loading of implants of 5–6 months. Bone condensing was performed to enhance the bone density.	Fixed and removable
Schultes et al. [15]	2002	Retrospective observational	Stability of implants in microvascular free flaps	Poorly defined - implant still in situ then survived	N/A	8/16	Alveolar crest carcinoma T4	Average 58.2, 53.6 years	Up to 12 months	Mandible	SIS (Austria)	Implants placed 4 months after radiotherapy Delayed loading of implants of 4 months	All removable
Buddula et al. [24]	2010	Retrospective observational	Implant survival in irradiated bone	Own—implant present in oral cavity at time of data collection then deemed to have survived.	N/A	13/16	SCC, adenoid cystic carcinoma, BCC and unknown	Mean = 60.2 years	Up to 7 years	Maxilla and mandible	Unknown	Median time from ending radiotherapy to implant placement was 3.4 years.	Unknown
Klein et al. [32]	2009	Retrospective observational	Prognostic parameters for the rehabilitation of mandibular continuity defects with free autologous bone and dental implants for patients after intra-oral squamous cell carcinoma	N/A	Naert et al. 1992	11/16	SCC	Mean = 55.7 years	Not documented	Mandible	Unknown	Implants were principally placed into the following 4 tissue conditions: non-irradiated local bone, irradiated local bone, osteoplastic in non-irradiated tissue and osteoplastic in irradiated tissue.	Unknown
Burgess et al. [27]	2017	Retrospective observational	Implant survival in a variety of composite free flaps	Own—implant not removed then survived	N/A	10/16	Head and neck neoplasia	Average age at implantation was 51 years (range, 18–77 years)	At least 6 months follow-up	Maxilla and mandible	Neoss, Straumann Dentsply Sirona, South Africa - Head Office implants	Primary and secondary implant placement. The mean time to implant placement from reconstruction was 19 months (range, 0–141 months) with 2 patients (7 implants) having their implants placed into the fibula 6 weeks before harvesting.	Unknown
Chiapasco et al. [18]	2006	Retrospective observational	Fibula Free flap survival, implant survival	Albrektsson et al.1986	Albrektsson et al.1986	9/16	Rhabdomyosarcoma, sarcoma, SCC, osteosarcoma and ameloblastoma	Range = 13–66 years	Range = 24–106 months	Maxilla and mandible	Branemark, ITI and 3i	Placement using surgical guides. Secondary implant placement 3–12 months after oncological reconstruction. Implants immediately loaded in 2 patients. Delayed loading for the all other patients 3-6 months after placement.	Fixed and removable
Chiapasco et al. [23]	2008	Retrospective observational	Bone graft success, implant success, patient satisfaction	Own—similar to Albrektsson et al.1986 authors allow greater bone loss around implants.	Albrektsson et al.1986	7/16	Ameloblastoma, ossifying fibroma, cementoblastoma, myxoma, SCC, gigantocellular tumour, OKC and rhabdomyosarcoma.	Range = 17–54 years	Range = 48–132 months	Mandible	Straumann, Nobel biocare and Branemark	Placement using surgical guides. Secondary implant placement 4–7 months after oncological reconstruction. Delayed loading of implants 4-6 months after placement.	All fixed
Chiapasco et al. [33]	2000	Retrospective observational	Bone resorption of bone grafts, behaviour of bone around implants, implant failure	Albrektsson et al.1986	Albrektsson et al.1986	10/16	Ewing sarcoma, epidermoid carcinoma, cylindroma, desmoplastic fybroma, chondroblastic sarcoma, cementoblastoma, ameloblastoma, chondrosarcoma, ossifying fibroma, myxoma and giantocellular tumour	Range = 20–58 years	Range = 14–34 months	Maxilla and mandible	Branemark and ITI	Placement using surgical guides. Secondary implant placement 4–8 months after oncological reconstruction. Delayed loading of implants 4-6 months after placement.	Unknown
Hessling et al. [28]	2015	Retrospective observational	Implant survival, peri-implantitis	Poorly defined—implant still in situ then survived.	N/A	8/16	SCC and odontogenic tumours with malignant degeneration	Range = 18–77 years	Range = 3–82 months	Maxilla and mandible	Xive and templant	No described protocol.	Fixed and removable

The characteristics and MINORS (Methodological Index for Non-Randomized Studies) score for each of the 20 studies included within the review divided into those studies with a mean follow-up of 3 years or greater and those studies with a mean follow-up of less than 3 years or where no mean follow-up was reported within the study

Those marked with an asterisk have had the survival percentages calculated by the authors due to their being adequate information/data within the studies to calculate this

*Abbreviations*: *SCC* squamous cell carcinoma, *BCC* basal cell carcinoma, *OKC* odontogenic keratinocyst, *ACC* adenoid cystic carcinoma, *RDX* radiotherapy, *chemo* chemotherapy, *ORN* osteoradionecrosis, *DCIA* deep circumflex iliac artery flap, *MINORS* Methodological Index for Non-Randomized studies

### Statistical analysis

Due to the lack of controlled studies and the heterogeneity of the studies concerning patient selection, surgical protocols, implant loading, follow-up and prosthetic rehabilitation, implant survival definitions and figures, measurement protocols, and inconsistency in data reporting a formal meta-analysis would be statistically inappropriate and was not conducted. Descriptive statistics where used to interpret and present the data from these studies.

### Results of the studies

Descriptive data extraction was carried out for the 20 studies and is summarised in Tables 1 and 2. All studies were retrospective observational studies in design with the majority undertaken at single centres; however, for 3 studies, this was unclear (Schultes et al. [15], Yerit et al. [16], Linsen et al. [17]). These 20 studies were published over a range of 21 years (1996 to 2017) and provide cumulative data on 1905 implants placed into autogenous bone grafts in H&N cancer patients with both benign and malignant tumours being reported. The exact patient number for this intervention within some of the studies was unclear as a result of the studies reporting on implant rather than patient number or there was an inability to identify which population received dental implants to identify patient numbers. One study (Chiapasco et al. [18]) included reported on patients under 18 years old (two patients in total); however, these patients and their data could be removed from the analysis.

**Table 2:** Summary of implant survival and implant success in autogenous bone grafts

					Implant survival				Implant success				
Author	Year of publication	Donor site of autogenous bone graft	Radiotherapy/chemotherapy to bone graft site	Complications									
					No. of patients who had implants placed into autogenous bone grafts (and failures)	Overall patient implant survival in autogenous bone grafts	No. of implants placed into autogenous bone grafts (and failures)	Overall implant survival in autogenous bone grafts	No. of patients who had implants placed into autogenous bone grafts (and unsuccessful)	Overall patient implant success in autogenous bone grafts	No. of implants placed into autogenous bone grafts (and unsuccessful)	Overall implant success in autogenous bone grafts	Reasons for a lack of implant success
Studies with an average follow-up of 3 years or greater													
Watzinger et al. [29]	1996	Vascularised iliac bone graft and non-vascularised iliac and rib bone graft	Yes—all patients had chemotherapy and RDX	Marginal bone loss, periodontal pocketing, gingival index and sulcus bleeding index showed wide variation	Not reported	N/A	52 (14)	73.1%*	Not reported	N/A	52 (22)	57.7%*	Non-functioning implants (not prosthetically loaded)
Teoh et al. [26]	2005	Vascularised fibula free flap	Yes—5 patients had chemotherapy, 1 patients had chemo/RDX (pre-implant placement), 6 patients had pre-op RDX and 1 patient had post-op RDX.	13 patients had soft tissue hyperplasia that need debulking or skin grafting	22 (2)	90.9%*	71 (3)	95.8%*	Not reported	N/A	Not reported	N/A	N/A
Wu et al. [30]	2008	Fibula free flap	Yes—3 patients had RDX (unsure if pre or post-op)	Soft tissue hyperplasia needed surgical removal in 6 patients (17 implants).	29 (not reported)	N/A	100 (9)	91.0%	29 (not reported)	N/A	100 (14)	86.0%	Unfavourable local soft tissue and implant left as sleepers. Peri-implant bone loss greater than 2 mm
Fenlon et al. [19]	2012	Vascularised free flap—DCIA, radial, fibula and rib	Yes—35 implants had RDX	High rate of poor implant positioning in primary implant placement.	41 (10)	75.6%*	145 (18)	87.5%*	Not reported	N/A	145 (34)	76.6%*	Implants osseointegrated but prosthetically unusable
Ch’ng et al. [20]	2014	Vascularised fibula free flap	Yes −66/243 patients had RDX (43 patients pre-op RDX, 23 patients post-op RDX)	ORN 7.7% of all implants (19 patients, 4 cases in vascularised fibula free flap and 15 in native bone) smoking was shown to be a significant risk factors. Also modification of peri-implant soft tissue required such as debulking of soft tissue and vestibuloplasty as required.	54 (10)	81.5%*	243 (20)	91.8%	Not reported	N/A	Not reported	N/A	N/A
Shaw et al. [31]	2005	Vascularised composite DCIA, fibula and radius and non-vascularised bone grafts	Yes—47% of patients had RDX	Soft tissue overgrowth in 3 patients (5 implants). Also, surgical debulk of soft tissue reported in number of cases.	33 (12)	63.6%*	123 (32)	69.0%	Not reported	N/A	Not reported	N/A	N/A
Wang et al. [21]	2015	Vascularised fibula free flap (double barrel or vertical distraction osteogenesis techniques)	NO	Implant hygiene and bleeding increased over time. 6 patients (11 implants) required soft tissue reduction however recurrence of soft tissue overgrowth occurred.	19 (0)	100%	51 (0)	100%*	Not reported	N/A	51 (7)	86.3%*	Peri-implant bone loss greater than criteria (radiographic assessment)
Yerit et al. [16]	2006	Vascularised and non-vascularised iliac bone graft	No—No RDX to bone graft sites	None noted only documenting causes of implant loss	Not reported	N/A	78 (13)	54.0%	Not reported	N/A	Not reported	N/A	N/A
Linsen et al. [17]	2009	Avascularised iliac bone graft	Yes—39 implants had RDX, 44 implants did not have RDX	Peri-implantitis in 12 patients (31 implants).	Not reported	N/A	79 (8)	89.9%*	Not reported	N/A	Not reported	N/A	N/A
Studies with an average follow-up of less than 3 years or no average follow-up reported													
Fierz et al. [25]	2013	Vascularised free flap—fibula, radius, scapula	Yes—20 out of 46 implants had RDX	Frail patients limited treatment, and prosthetic rehabilitation was challenging	Not reported	N/A	46 (8)	82.6%*	Not reported	N/A	Not reported	N/A	N/A
Barrowman et al. [7]	2011	Vascularised free flap—illiac, DCIA and fibula and non-vascularised bone graft.	Yes—15 implants in to irradiated vascularised free flap	Inability of patients to tolerate prosthesis. Peri-implantitis and lack of integration of some implants.	Not reported	N/A	38 (5)	86.8%*	Not reported	N/A	Not reported	N/A	N/A
Zou et al. [22]	2013	Vascularised iliac bone graft	No	Increase in plaque index over time. Prosthodontic complications overtime after prosthesis fitted also tumour recurrence	32 (not reported)	N/A	110 (4)	96.4%	Not reported	N/A	110 (9)	91.8%	Severe gingival hyperplasia and bone resorption in peri-implant area
Schultes et al. [15]	2002	Vascularised scapula and iliac bone graft	Yes—all patients had RDX 60 Gys.	Increased pocket depth around implants placed into non-native bone in comparison to native bone. 7 implants with pocketing greater than 5 mm were all in vascularised free flaps	38 (2)	94.7%*	96 (2)	97.9%*	Not reported	N/A	96 (4)	95.8%*	Implants inadequately positioned and could not be used for further prosthetic treatment
Buddula et al. [24]	2010	Bone graft—fibula, iliac and scapula (unsure of vascularised or non-vascularised)	Yes—all patients had RDX	None noted only documenting implant survival	Not reported	N/A	59 (8)	83.3%	Not reported	N/A	Not reported	N/A	N/A
Klein et al. [32]	2009	Avascular iliac bone graft	Yes—some patients had RDX	None noted only documenting implant survival	Not reported	N/A	128 (22)	78.4%	Not reported	N/A	Not reported	N/A	N/A
Burgess et al. [27]	2017	Vascularized bone grafts—fibula, DCIA, scapula and radial	Yes—some patients had RDX	None noted only documenting implant survival	59 (not reported)	N/A	199 (11)	93.6%	Not reported	N/A	Not reported	N/A	N/A
Chiapasco et al. [18]	2006	Vascularised fibula free flap	Yes—some patients had RDX and chemo—unknown number	Soft tissue overgrowth in 2 patients that required removal and palatal mucosal graft placed	14 (1)	92.9%*	62 (1)	98.3%*	14 (2)	85.7%*	62 (5)	91.9%*	Peri-implant bone loss greater than criteria (radiographic assessment)
Chiapasco et al. [23]	2008	Non-vascularised—Calvarium or iliac bone graft	Unknown	Soft tissue grafting required around implants in 3 patients	16 (1)	93.8%*	60 (2)	96.7%	16 (2)	87.5%*	60 (4)	93.3%	Peri-implant bone loss greater than criteria (radiographic assessment)
Chiapasco et al. [33]	2000	Non-vascularised—ilieum and fibula, and vascularised free flap—ilieum and fibula	Yes—3 patients had RDX (unknown if pre or post)	Soft tissue grafting required around implants in 3 patients	18 (2)	88.9%*	72 (3)	95.8%*	18 (2)	88.9%*	72 (3)	95.8%*	N/A
Hessling et al. [28]	2015	Free iliac crest, microvascular iliac, microvascular fibula, microvascular scapula, calavarial bone graft	Yes—some patients had RDX and chemo (pre- and post-op) unknown number	67% peri-implantitis due to a lack of attached gingivae	Not reported	N/A	93 (8)	91.4%*	Not reported	N/A	Not reported	N/A	N/A

Implant survival and implant success in autogenous bone grafts was extracted on a patient and implant level (where applicable) for all 20 studies included within this review

Those marked with an asterisk have had the survival/success percentages calculated by the authors due to their being adequate information/data within the studies to calculate this

*Abbreviations*: *RDX* radiotherapy, *chemo* chemotherapy, *DCIA* deep circumflex iliac artery flap, *pre-op* pre-operative, *post-op* postoperative

Implants were placed into both vascularised and non-vascularised autogenous bone grafts, with a number of donor sites being reported. (Tables 2 and 3) These implants were placed in a variety of intra-oral sites with implants placed into autogenous bone grafts within the mandible reported in eight studies and bi-maxillary placement in nine studies, and in three studies, it was unknown where the implant fixtures were placed other than that they were placed into autogenous bone grafts (Linsen et al. [17], Fenlon et al. [19], Ch’ng et al. [20]). There were no studies where implants were placed solely in the reconstructed maxilla.

**Table 3:** Implant survival in autogenous bone grafts placed in vascularised and non-vascularised bone grafts

		Non-vascularised bone graft				Vascularised bone graft			
Author	Year of publication	No. of patients who had implants placed into non-vascularised autogenous bone grafts (and failures)	Overall patient implant survival in non-vascularised autogenous bone grafts	No. of implants placed into non-vascularised autogenous bone grafts (and failures)	Overall implant survival in non-vascularised autogenous bone grafts	No. of patients who had implants placed into vascularized autogenous bone grafts (and failures)	Overall patient implant survival in vascularised autogenous bone grafts	No. of implants placed into vascularised autogenous bone grafts (and failures)	Overall implant survival in vascularised autogenous bone grafts
Studies with an average follow-up of 3 years or greater									
Watzinger et al. [29]	1996	Not reported	N/A	33 (13)	60.6%*	Not reported	N/A	19 (1)	94.7%*
Teoh et al. [26]	2005	N/A	N/A	N/A	N/A	22 (2)	90.9%*	71 (3)	95.8%*
Wu et al. [30]	2008	N/A	N/A	N/A	N/A	29 (not reported)	N/A	100 (9)	91%
Fenlon et al. [19]	2012	N/A	N/A	N/A	N/A	41 (10)	75.6%*	145 (18)	87.5%*
Ch’ng et al. [20]	2014	N/A	N/A	N/A	N/A	54 (10)	81.5%*	243 (20)	91.8%
Shaw et al. [31]	2005	2 (1)	50%*	8 (2)	75%*	31 (11)	64.5%*	115 (30)	73.9%*
Wang et al. [21]	2015	N/A	N/A	N/A	N/A	19 (0)	100%	51 (0)	100%*
Yerit et al. [16]	2006	Not reported	N/A	Not reported	N/A	Not reported	N/A	Not reported	N/A
Linsen et al. [17]	2009	Not reported	N/A	79 (8)	89.9%*	N/A	N/A	N/A	N/A
Studies with an average follow-up of less than 3 years or no average follow-up reported									
Fierz et al. [25]	2013	N/A	N/A	N/A	N/A	Not reported	N/A	Not reported	N/A
Barrowman et al. [7]	2011	Not reported	N/A	6 (0)	100%*	Not reported	N/A	32 (5)	84.4%*
Zou et al. [22]	2013	N/A	N/A	N/A	N/A	32 (not reported)	N/A	110 (5)	96.4%
Schultes et al. [15]	2002	N/A	N/A	N/A	N/A	38 (2)	94.7%*	96 (2)	97.9%*
Buddula et al. [24]	2010	Not reported	N/A	Not reported	N/A	Not reported	N/A	Not reported	N/A
Klein et al. [32]	2009	Not reported	N/A	128 (22)	82.8%*	N/A	N/A	N/A	N/A
Burgess et al. [27]	2017	N/A	N/A	N/A	N/A	59 (not reported)	N/A	199 (11)	93.6%
Chiapasco et al. [18]	2006	N/A	N/A	N/A	N/A	14 (1)	92.9%*	62 (1)	98.3%*
Chiapasco et al. [23]	2008	16 (1)	93.8%*	60 (2)	96.7%*	N/A	N/A	N/A	N/A
Chiapasco et al. [33]	2000	10 (1)	90%*	41 (2)	95.1%*	8 (1)	87.5%*	31 (1)	96.8%*
Hessling et al. [28]	2015	Not Reported	N/A	62 (4)	93.5%*	Not reported	N/A	31 (4)	87.1%*

Implant survival in autogenous bone grafts was extracted on a patient and implant level (where applicable) for all 20 studies included within this review that specifically reported on implant survival in either vascularised or non-vascularised autogenous bone grafts

Those marked with an asterisk have had the survival percentages calculated by the authors due to their being adequate information/data within the studies to calculate this

Radiotherapy to the autogenous bone graft/implant site was reported in 16 studies. Two studies (Wang et al. [21], Zou et al. [22]) reported that radiotherapy was not carried out on the study population and in 1 study (Yerit et al. [16]) bone graft sites were not irradiated. One study (Chiapasco et al. 2008 [23]) failed to report whether radiotherapy was carried out or not on the study population. Of 20 studies included in the systematic review, only 7 studies reported on outcomes related to implant survival in irradiated autogenous bone grafts (Barrowman et al. [7], Fenlon et al. [19], Ch’ng et al. [20], Buddula et al. [24], Fierz et al. [25], Teoh et al. [26], Burgess et al. [27]).

The surgical and loading implant protocols were reported in 17 studies with no description given in 3 studies (Barrowman et al. [7], Fierz et al. [25], Hessling et al. [28]). The implant placement protocols were diverse with variables including the use of surgical templates/guides, primary and/or secondary implant placement following autogenous bone grafting, and immediate and/or delayed implant loading; however, the majority of the studies reported on delayed implant placement following initial healing of the transported bone graft and delayed loading of the implant fixtures. Six studies reported primary implant placement (Fenlon et al. [19], Ch’ng et al. [20], Zou et al. [22], Burgess et al. [27], Wu et al. [30], Watzinger et al. [29],) and one study reported immediate implant loading (Chiapasco et al. [18]). Additional procedures were also reported which include removal of reconstruction plates and screws at the time of implant placement, bone condensing to enhance the bone density, and further peri-implant surgery in the form of debulking of soft tissues, gingivoplasty/vestibuloplasty and free mucosal grafts to optimise the soft tissue conditions (Table 1). Prosthodontic reconstruction of the implant fixture was reported in 15 of the studies which included fixed and removable prosthesis and is summarised in Table 1.

#### Overall implant survival

The overall implant survival of implants placed into autogenous bone grafts varied highly (both at implant and patient levels) between the included studies ranging from 100% with a mean follow-up of 3.5 years ± 0.3 years in a study by Wang et al. [30] to 54% with a mean follow-up 5.4 years ± 3.2 years by Yerit et al. [16].,(at an implant level) (Table 2).

Eleven studies compared implant survival in autogenous bone grafts to that in native bone within their studies. Nine of these studies (Barrowman et al. [7], Yerit et al. [16], Linsen et al. [17], Fenlon et al. [19], Ch’ng et al. [20], Hessling et al. [28], Watzinger et al. [29], Shaw et al. [31], Klein et al. [32]) reported higher implant failure rates within autogenous bone grafts than those within implants placed into the native bone; however, two studies (Buddula et al. [24], Teoh et al. [26]) reported no significant difference.

#### Autogenous bone graft type and implant survival

Seventeen studies reported on the specific bone graft type (non-vascularised or vascularised) into which the implants were placed. In the remaining three studies (Buddula et al. [24], Fierz et al. [25], Yerit et al. [16]), this distinction was not possible.

Of these 17 studies, 8 studies reported on implant survival in non-vascularised bone grafts and 14 studies reported on implant survival in vascularised bone grafts with 5 studies (Barrowman et al. [7], Hessling et al. [28], Watzinger et al. [29], Shaw et al. [31], Chiapasco et al. [33]), therefore reporting on implant survival in both non-vascularised and vascularised bone grafts within their study (Table 3). Implant survival appears to be higher for those implants placed into vascularised bone grafts in comparison to non-vascularised bone grafts. Of the five studies reporting on both vascularised and non-vascularised bone grafts, three of these studies (Barrowman et al. [7], Watzinger et al. [29], Chiapasco et al. [33]) reported higher implant survival in vascularised bone grafts whereas the other two studies (Hessling et al. [28], Shaw et al. [31]) reported higher implant survival in non-vascularised bone grafts. Shaw et al. [31] reported that implants placed into ‘vascularized bone graft were superior to non-vascularized bone. In particular, those implants in composite radial forearm flaps performed badly. With the proportion of patients with implant loss in these bone flaps within their study being 27% in iliac crest, 33% in fibula, and 100% in radius and that implants placed in composite fibula and iliac crest flaps performed approximately as well as in native maxilla within their study’ [31].

Twelve studies reported on the use of more than one autogenous bone graft donor site within their study (Barrowman et al. [7], Schultes et al. [15], Yerit et al. [16], Fenlon et al. [19], Chiapasco et al. [23], Buddula et al. [24], Fierz et al. [25], Burgess et al. [27], Hessling et al. [28], Watzinger et al. [29], Shaw et al. [31] and Chiapasco et al. [33]); of these, five studies reported on the effect of the autogenous bone graft donor site on implant survival. Two studies (Fenlon et al. [19], Burgess et al. [27]) reported no significant effect on implant survival in varying graft donor sites; however, three studies (Hessling et al. [28], Shaw et al. [31], Chiapasco et al. [33]) reported varying implant survival rates within different autogenous bone grafts but only one study (Hessling et al. [28]) reported that implant loss was significant with this being for implants placed into fibula bone grafts. Shaw et al,. [31] reporting that implants placed into ‘vascularized bone graft were superior to non-vascularized bone. In particular, those implants in composite radial forearm flaps performed badly. With the proportion of patients with implant loss in these bone flaps within their study being 27% in iliac crest, 33% in fibula, and 100% in radius and that implants placed in composite fibula and iliac crest flaps performed approximately as well as in native maxilla within their study’ [31].

#### Radiotherapy and implant survival

Seven studies reported on outcomes related to implant survival in irradiated autogenous bone grafts (Barrowman et al. [7], Fenlon et al. [19], Ch’ng et al. [20], Buddula et al. [24], Fierz et al. [25], Teoh et al. [26], Burgess et al. [27]) (Table 4). One study reported solely on irradiated patients (Buddula et al. [24]) the other six studies (Barrowman et al. [7], Fenlon et al. [19], Ch’ng et al. [20], Fierz et al. [25], Teoh et al. [26], Burgess et al. [27]) reported on both irradiated and non-irradiated patients. These six studies (Barrowman et al. [7], Fenlon et al. [19], Ch’ng et al. [20], Fierz et al. [25], Teoh et al. [26], Burgess et al. [27]) all reported higher implant failure (at an implant and a patient level (where applicable)) of implants placed into autogenous bone grafts in irradiated patients in comparison to those patients who did not received radiotherapy (Table 4).

**Table 4:** Implant survival in autogenous bone grafts of irradiated & non-irradiated patients

		RDX				No RDX			
Author	Year of publication	No. of implants placed into autogenous bone grafts with RDX (and failures)	Overall implant survival of implants placed into autogenous bone grafts with RDX	No. of patients who had implants placed into autogenous bone grafts with RDX (and failures)	Patient based implant survival of implant placed into autogenous bone grafts with RDX	No. of implants placed into autogenous bone grafts with no RDX (and failures)	Overall implant survival of implants placed into autogenous bone grafts with no RDX	No. of patients who had implants placed into autogenous bone grafts with no RDX (and failures)	Patient-based implant survival of implant placed into autogenous bone grafts with no RDX
Teoh et al. [26]	2005	14(2)	85.7%*	4 (1)	75%*	57 (1)	98.2%*	22 (1)	95.4%*
Fenlon et al. [19]	2012	35 (15)	57.1%*	12 (8)	33.3%*	110 (3)	97.3%*	29 (2)	93.1%*
Ch’ng et al. [20]	2014	66 (11)	83.3%*	Not reported	N/A	177 (9)	94.9%*	Not reported	N/A
Fierz et al. [25]	2013	20 (6)	70.0%*	Not reported	N/A	26 (2)	92.3%*	Not reported	N/A
Barrowman et al. [7]	2011	15 (5)	66.7%*	Not reported	N/A	23 (0)	100%*	Not reported	N/A
Buddula et al. [24]	2010	59 (8)	83.3%	Not reported	N/A	N/A	N/A	N/A	N/A
Burgess et al. [27]	2017	45* (7)	84.4%*	Not reported	N/A	154 (4)	97.4%*	Not reported	N/A

Implant survival in autogenous bone grafts of irradiated and non-irradiated patients was extracted on an implant and patient level (where applicable) for seven studies that reported on implant survival of implants placed in autogenous bone grafts

Those marked with an asterisk have had the survival percentages calculated by the authors due to their being adequate information/data within the studies to calculate this

*Abbreviations*: *RDX* radiotherapy

All of these studies (Barrowman et al. [7], Fenlon et al. [19], Ch’ng et al. [20], Fierz et al. [25], Teoh et al. [26], Burgess et al. [27]) reported on the deleterious effect of radiotherapy on implant survival in autogenous bone grafts within their studies and was found to be statistically significant in two studies (Fenlon et al. [19], Ch’ng et al. [20]) with Fenlon [19] reporting a close correspondence of implant survival (in vascularised free composite grafts) and an absence of radiotherapy using a multiple correspondence analysis and Ch’ng et al. [20] who reported a statistical significance associated with higher implant failure in irradiated fibula free flaps in comparison to non-irradiated fibula free flaps (*P* = 0.041). However, in two studies (Teoh et al. [26], Burgess et al. [27]), no statistical significance was found despite higher implant failure.

#### Primary and secondary implant placement and implant survival

Six studies clearly reported the use of both primary and secondary implant placement within their study (Fenlon et al. [19], Ch’ng et al. [20], Zou et al. [22], Burgess et al. [27], Watzinger et al. [29], Wu et al. [30]); however, only one study (Fenlon et al. [19]) reported on implant survival in primary and secondary implant placement within autogenous bone grafts. Felon et al. [19] reported on implant survival in immediate vs delayed placement of the implant fixtures into free vascularised grafts and found that implant survival of immediately placed implants was significantly worse than that of implants placed after a delay of 3 months in free vascularized grafts.

#### Cancer diagnosis and implant survival

With regards to cancer type (malignant vs benign), three studies (Schultes et al. [15], Watzinger et al. [29], Klein et al. [32]) reported exclusively on implant survival in patients with malignant H&N cancers with varying implant survival rates being reported, whilst one study reported exclusively on benign H&N cancer patients (Wang et al. [21]) with a 100% implant survival rate being reported (Table 2). Two studies (Fenlon et al. [19], Burgess et al. [27]) provided non-descriptive terms (cancer, head and neck neoplasia) for the type of H&N cancer of the patients within their studies and therefore differentiation between benign and malignant disease could not be made. The other 14 studies reported on both malignant and benign H&N cancers; however, the implant survival data was not reported or presented in a way in which comparison of implant survival in patients with malignant or benign H&N cancers could be made.

#### Implant survival and Peri-implant soft tissue

Only one study (Linsen et al. [17]) reported on the effect of the peri-implant soft tissue and implant survival of implants placed into autogenous bone grafts. Linsen et al. [17] reported a higher implant failure of implants placed into bone and soft tissue grafts in comparison to implants placed into a bone grafts with residual soft tissues. This difference, however, was not found to be statistically significant (*p* = 0.436).

In the other 19 studies, the effect of the peri-implant soft tissue was not directly reported as being a factor for implant survival. However, implant success appeared to be significantly affected by the peri-implant soft tissues (see the “Implant survival and Implant Success” and “Complications” sections – for further details).

#### Implant survival and implant success

In nine studies *(*Schultes et al. [15], Fenlon et al. [19], Wang et al. [21], Zou et al. [22], Chiapasco et al. [18], Chiapasco et al. [23], Watzinger et al. [29] Wu et al. [30], Chiapasco et al. [33]), both implant survival and success data was reported or provided (Table 2). When comparing implant survival and implant success in eight studies *(*Schultes et al. [15], Fenlon et al. [19], Wang et al. [21], Zou et al. [22], Chiapasco et al. [18], Chiapasco et al. [23], Watzinger et al. [29], Wu et al. [30], Chiapasco et al. [33]) implant success was found to be lower than implant survival but in one study (Chiapasco et al. [33]) implant survival and success were reported as being the same. The reasons for a lack of implant success within these eight studies (other than implant failure/loss) were related to excessive peri-implant bone loss in five studies (Wang et al. [21], Zou et al. [22], Chiapasco et al. [18], Chiapasco et al. [23], Wu et al. [30]), an inability to prosthetically restore the implants in four studies *(*Schultes et al. [15], Fenlon et al. [19], Watzinger et al. [29], Wu et al. [30]) and gingival hyperplasia in one study (Zou [22]). Six of these studies (Schultes et al. [15], Wang et al. [21], Zou et al. [22], Chiapasco et al. [18], Chiapasco et al. [23], Wu et al. [30]) reported some of this lack of success to the peri-implant soft tissue which was most frequently the soft tissue component of a combined bone and soft tissue free flap (most commonly the external skin).

#### Complications

A variety of implant-based complications were documented. Complications were often described within the study rather than being formal assessed, defined or used as outcome measures. Due to there being a lack of formal definition and variability in the documentation within the studies, the data cannot be considered robust to be collectively appraised but is described for information purposes. Common “complications” reported in the studies include soft tissue overgrowth/hyperplasia of the peri-implant tissues (Wang et al. [21], Chiapasco et al. [18], Teoh et al. [26], Wu et al. [30], Shaw et al. [31]), peri-implantitis and periodontal pocketing (Barrowman et al. [7], Schultes et al. [15], Linsen et al. [17], Burgess et al. [27], Hessling et al. [28]), the need for soft tissue debulking/modification around free flaps (Ch’ng et al. [20], Shaw et al. [31]) and the need for mucosal/soft tissue graft around implants to improve the soft tissue profile (Chiapasco et al. [23], Teoh et al. [26], Chiapasco et al. [33]). These peri-implant complications were most commonly seen when the soft tissue profile around the implant was related to a soft tissue graft and therefore did not have attached keratinised mucosa which is needed to provide a soft tissue profile that is conducive to peri-implant health. Other complications include poor oral hygiene (Wang et al. [21], Zou [22]), challenging prosthodontic rehabilitation/inability to tolerate the prosthesis provided (Barrowman et al. [7], Zou et al. [22], Fierz et al. [25]), poor implant position (Schultes et al. [15], Fenlon et al. [19], Watzinger et al. [29], Wu et al. [30]) and osteoradionecrosis (Ch’ng et al. [20]) (Table 2).

## Discussion

### Summary of evidence

Dental implants are now perceived to be a vital part of the clinician’s armamentarium in the provision of oral and dental rehabilitation for patients with acquired deformity following management of their H&N cancer, and therefore, this systematic review is relevant to clinicians and stakeholders involved in the treatment and management of H&N cancer patients specifically with those involved in placing or utilising dental implants to assist in the dental/oral rehabilitation of H&N cancer patients.

The main findings from this systematic review did however identify, with the exception of a small number of studies, implant survival (at an implant level) in autogenous bone grafts was clinically promising (> 85%); however, this appears to be lower than implants placed into the native bone in H&N cancer patients. Weak evidence was identified which suggests that radiotherapy is a prognostic factor affecting implant survival in this patient cohort; however, this has also been reported as having a detrimental effect on implant survival in the native bone within the literature [34]. The type of autogenous bone graft donor site and implant survival was also reviewed within the included studies that compared varying autogenous bone graft donor sites and implant survival. There is some weak evidence from these studies to suggest that implants placed into vascularised bone grafts appear to have a higher survival rate in comparison to non-vascularised bone grafts within this review. This evidence however is unreliable, due to the clear lack of studies reporting on implant survival in non-vascularised bone grafts and thus the subsequent number of implants and patients included within this review. Implant survival did not appear to be affected by the type of H&N cancer type (malignant vs. benign); however, no studies within this review directly compared or enabled the authors of this manuscript to compare studies, and accordingly, no true conclusion can be made on this.

The implant placement protocol with regard to primary (immediate) or secondary (delayed) implant placement was also reviewed, and there is limited evidence from Fenlon et al. that implant failure is significantly worse in immediately placed implants in comparison with a delayed approach in free vascularized grafts.

Implant success was shown to be lower than implant survival and was related to peri-implant bone loss, peri-implant hyperplasia and an inability to prosthetically restore the implants. This was most commonly related to combined bone and soft tissue grafts, specifically the soft tissue component. This soft tissue component provides a suboptimal soft tissue profile which could contribute to implant failure (as a result of peri-implantitis); however, well-designed long-term studies are needed to fully comprehend the effect on implant survival.

Implant complications were also noted specific to autogenous bone grafts related to peri-implant soft tissue overgrowth/hyperplasia and the possible need for soft tissue debulking/modification and mucosal/soft tissue graft around implants, which occurred commonly in combined bone and soft tissue grafts. These finding, however, are limited to low-level evidence in the form of a small number of retrospective observational studies.

### Limitations

This systematic review has identified that the quality of evidence to inform clinical decision making regarding the use of implants in transported bone in this patient group is currently deficient. All studies included in the review were retrospective observational studies and in general reported on low patient and implant number and found to be at moderate to serious risk of bias.

A lack of consistency in definitions of the primary (implant related) outcome measures was observed. The outcome measures used in the studies varied and implant survival/success was not necessarily the primary outcome measure. Only 14 of the 20 studies reported the primary outcome measure to be implant survival/success whilst the remainder reported free flap survival, graft success and bone resorption of bone grafts as the primary outcome.

A clear deficiency of many of the studies was the imprecise and inconsistent definitions of implant survival or implant success, as detailed in Table 1. In addition, in a number of studies, the terminology ‘implant success’ and ‘implant survival’ were used interchangeably within the narrative making comparison of the studies challenging and rendering statistical analysis of the survival data inappropriate.

The reporting of implant survival data varied between studies and was presented in a variety of ways which included cumulative survival and implant survival incidence. In some cases, no attempt to estimate survival was made but adequate data was documented to enable its calculation (Table 2). Best practice would be the reporting of cumulative survival to give context to survival (time) and account for patient drop-out which may be high in this particular patient group. Due to the variability in the methods of data reporting and their comprehensiveness, there was insufficient confidence in extracted data to report statistical findings. Notably, as all studies presented different deficiencies in data reporting or study definitions, there was no clear way to further exclude studies using these criteria.

As such, there is a clear need for a consensus on what minimum data set is required for published articles reporting on implant survival in this patient cohort to allow further investigation via systematic reviews (e.g., effect of benign vs malignant H&N cancer and implant survival). The inclusion and exclusion criteria were highly variable, and in some studies, the criteria were such that there was a pre-disposition to selection bias and reporting higher implant survival rates. Patient follow-up was variable and also variably reported but in general was insufficient. Where possible, follow-up of at least 5 years is required to begin to evaluate the outcome of dental implant treatment. Unfortunately, information on long-term dental survival in this cohort is still scarce and the results of the present review should not be extrapolated beyond early implant survival.

## Conclusion

Within the limitations of the current review, it can be concluded that implant survival in autogenous bone grafts in H&N oncology patients appears to be promising with implant survival being reported at over 80% in 16 of the 20 studies included with 11 of these reporting implant survival of over 90% in follow-up ranging from 3 months [28] to 15 years [5]. However, there is a lack of good quality evidence in the way of prospective studies and randomised control trials. A lack of long-term survival studies with sufficient implant and patient numbers was identified, and therefore, the results of the present review should not be extrapolated to longer follow-up times. Prognostic factors affecting implant survival in autogenous bone grafts were also reviewed with higher implant failure in autogenous bone grafts being reported in implants placed into irradiated autogenous bone grafts. Weak evidence suggesting implant failure was higher in non-vascularised in comparison with vascularised autogenous bone grafts and that implant failure was greater in primary placed implants in vascularised bone grafts in this cohort was identified. Implant success was lower than implant survival and was most commonly related to peri-implant disease and an inability to prosthetically to restore the implant. This was predominantly related to unfavourable peri-implant soft tissue which is frequently found around implants placed into combined bone and soft tissue flaps.

In order to understand the use of implants in autogenous bone grafts in H&N oncology patients larger, well-designed prospective studies are required. There needs to be clear set definitions of implant survival and success and appropriate presentation and statistical analysis of the data so that studies can be brought together to enable meta-analysis.

## Abbreviations

H&N: Head and neck
MINORS: Methodological Index for Non-Randomized Studies
PRISMA: Preferred reporting items for systematic reviews and meta-analyses
QoL: Quality of life
